# Immunogenic potential of a *Salmonella* Typhimurium live vaccine for pigs against monophasic *Salmonella* Typhimurium DT 193

**DOI:** 10.1186/s12917-017-1271-5

**Published:** 2017-11-17

**Authors:** Tobias Theuß, Elke Ueberham, Jörg Lehmann, Thomas Lindner, Sven Springer

**Affiliations:** 1 IDT Biologika GmbH, Business Unit Animal Health, Research and Development, Am Pharmapark, 06861 Dessau-Rosslau, Germany; 2 Fraunhofer Institute for Cell Therapy and Immunology, Perlickstraße 1, 04103 Leipzig, Germany

**Keywords:** Salmonella Typhimurium, Vaccination, Infection, Salmoporc, IgM, IgA, IgG, Serology, ELISA, Antibody response

## Abstract

**Background:**

Monophasic *Salmonella* Typhimurium (mSTM) strains account for up to 8.6% of all human Salmonellosis cases. They have an increasing prevalence during recent years and several human cases with hospitalisation were reported. These strains are often isolated from pigs and pork - one primary source of human infection. A *Salmonella* Typhimurium (STM) live vaccine has been proven successful in controlling of STM infections in pigs for many years. The aim of this study was to test the immunogenicity of the vaccine in weaners during oral challenge with a virulent mSTM strain and to examine the kinetics of STM-specific IgA, IgM and IgG antibodies induced by vaccination and infection.

**Results:**

Despite clinical signs being present in both groups, the vaccination led to a significant reduction of diarrhoea, overall clinical symptoms and a milder elevation of the body temperature. Necropsy revealed fewer pathological lesions in the gastrointestinal tract of vaccinated compared to control animals. Moreover, in the ileal and caecal mucosa and in the ileocaecal lymph nodes the challenge strain burden was significantly reduced by vaccination. Significant differences in the antibody responses of both groups were present during the vaccination period and after infection. In vaccinated animals *Salmonella*-specific IgA and IgG antibody levels increased significantly after vaccination and were even more pronounced in response to challenge. In contrast, similarly low levels of IgM antibodies were detected during the vaccination period in both vaccinated and non-vaccinated animals. However, after challenge IgM antibody levels increased significantly in control pigs while neither IgA nor IgG antibodies were detectable.

**Conclusion:**

The data demonstrate that mSTM can evoke clinical signs in weaners. Due to the vaccination their incidence and magnitude were significantly milder. Vaccination also led to a significantly reduced challenge strain burden in the intestine and the lymph nodes which is comparable to previous studies using the same vaccine in a challenge with biphasic STM. Therefore, it is concluded that this vaccine induces immunity against monophasic and biphasic STM strains. Furthermore, the results of antibody profiles in response to vaccination and infection provide additional evidence for humoral immune mechanisms triggered during *Salmonella* infection or vaccination.

## Background

*Salmonella* Enteritidis (SE), *Salmonella* Typhimurium (STM) and monophasic *Salmonella* Typhimurium strains (mSTM) were the most common reported serovars detected in human foodborne salmonellosis in the EU during recent years [1]. While confirmed infections with SE showed a decreasing incidence within the last years, those with STM and mSTM are progressively taking their place. In 2014 and 2015, 25.2% or 28.8% of all diagnosed cases of human Salmonellosis were attributed to these strains, respectively [1].

Monophasic *Salmonella* Typhimurium are often isolated from pigs and pork [1, 2]. Normally, they are multidrug-resistant and belong to phage type DT 120 and DT 193 (Anderson) [2]. In contrast to biphasic STM (seroformula 4,[5],12:i:1,2) their monophasic variants are lacking the H-antigen (seroformula 4,[5],12:i:-) according to the Kauffmann-White-LeMinor-Scheme [2]. Several diffuse outbreaks involving human cases with requirement of hospitalization have been reported [3–5].

Besides hygienic procedures at the barn level a STM live vaccine (Salmoporc®/Salmoporc STM®, IDT Biologika) based on an attenuated STM strain has been proven successful in controlling of STM infections in pigs for many years as shown in several laboratory [6–9] and field trials [10]. However, all of the mentioned laboratory studies have shown protection against STM. Protection against mSTM has not been confirmed in laboratory studies so far but is very likely upon consideration of the results of a large field study in the UK [10].

Although both humoral and cell-mediated immune mechanisms play an important role in the control of *Salmonella* infections [11], the detection of antibodies in blood serum or meat juice is of special interest as serology was and still is used in several governmental surveillance programs (e.g. Denmark, Belgium, Germany, Ireland, Sweden, The Netherlands).

Several commercial ELISA kits were developed but two different approaches dominated. Either an ELISA, based on a mix of LPS-antigen that is directed against *Salmonella*-specific IgG (Salmotype® PigScreen, Labor Diagnostik Leipzig, Leipzig, Germany; Swine Salmonella Ab Test, IDEXX, Hoofddorp, The Netherlands) or a test based on a whole-cell lysate of purified STM (Salmotype® Pig STM-WCE ELISA, Labor Diagnostik Leipzig) [12]. The latter test also allowed discrimination between *Salmonella*-specific IgM, IgA, and IgG [12]. Although this ELISA kit is no longer commercially available, it can be applied as an in-house format in our laboratory.

Numerous studies aimed to analyse the antibody response after STM infection [13–15] or vaccination [16]. However, to the best of our knowledge no approaches have been conducted to study the serological response of vaccination and subsequent challenge infection with STM in pigs.

Therefore, the aim of this study was to test the immunogenic potential of the vaccine against mSTM and to investigate the kinetic of antibody response to immunization and infection induced by the vaccine or the challenge strain, respectively.

## Methods

Weaners at the age of 4 weeks (*n* = 16, dam: German Landrace x German Large White, sire: Pietrain) were randomly selected from 4 sows and included in this study. All animals derived from the livestock of IDT Biologika GmbH and were owned by the company. The weaners were serologically negative for *Salmonella* spp. (< 10 OD %) and were tested bacteriologically negative for *Salmonella* spp. (individual faecal samples) prior to the beginning of the trial. All weaners were randomly selected to either the vaccination or the control group (*n* = 8 per group), which were housed separately in air-conditioned high-security rooms (BSL 2). The weaners were allocated in groups of 4 animals. Food and water were provided ad libitum and a commercial diet without anti-*Salmonella* ingredients (e.g. probiotics, prebiotics) was fed. The animal trial was conducted according to the German law of animal welfare (Reference no. 42502–3-753 IDT, Landesverwaltungsamt Sachsen-Anhalt). The study was performed as a randomized and blinded trial.

The vaccination group received 1.0 ml of the live attenuated *Salmonella* Typhimurium vaccine (Salmoporc®/Salmoporc STM®, IDT Biologika, Dessau-Rosslau, Germany) adjusted with physiologic saline solution at the minimal dose of 5 × 10^8^ CFU/ml. The vaccine strain was attenuated by chemical induced mutagenesis followed by phenotypical selection and is adenine/histidine auxotrophic. The control group received physiologic saline solution as placebo. The animals were vaccinated twice orally at an interval of 3 weeks using an oral drencher kit (IDT Biologika, Dessau-Rosslau, Germany) (study days 0 and 21). For drenching a second person fixed the animal in a slight upright position prior administration of the vaccine/placebo in the buccal cavity.

Three weeks after the 2nd administration of the vaccine (study day 42), both groups were orally infected with 5 × 10^9^ CFU/ml of a virulent *S*. *enterica* 4,[5],12:i: (DT 193) wild-type strain (kindly provided by Dr. W. Rabsch, National Reference Centre for *Salmonella* and other Enteric Bacterial Pathogens, Robert Koch Institute, Wernigerode, Germany) resistant to ampicillin (A), streptomycin (S), sulfamerazine (Su) and oxytetracycline (T). The challenge strain was administered to each pig in 5.0 ml sugar solution using an oral drench as described above.

Clinical scores were recorded daily during the challenge period according to the following scheme:

(i) diarrhoea (0 = none, 1 = yes, pulpy consistency, 2 = yes, liquid consistency)
(ii) depression (0 = none, 1 = reduced alertness, 2 = animal apathetic)
(iii)  food intake (0 = normal, 1 = reduced, 2 = no food intake)
(iv)  body temperature (in °C)

Summative ‘diarrhoea scores’ and ‘clinical scores’ (all scores added, including diarrhoea) were estimated daily for every group.

All animals were euthanized 6 or 7 days post challenge (p.chall.; study days 48 or 49, respectively; 4 animals of each group per day) by intravenous pentobarbital administration (Release® 500, WDT, Garbsen, Germany) during ketamine and azaperone anesthesia (Ursotamin®, Serumwerk Bernburg, Bernburg, Germany; Stresnil®, Elanco, Bad Homburg, Germany).

The quantitative determination of the number of challenge strain organisms (log_10_ CFU/g) of the ileal or caecal mucosa, and the ileocaecal lymph nodes was carried out using the Koch spread-plate method as previously described [6]. In brief, after weight assessment the tissue samples were homogenized (Ultra-Turrax T25, Janke & Kunkel, IKA®-Labortechnik Staufen, Germany) and a 10-fold dilution series was prepared. The number of bacteria was then estimated by plating out the homogenate on desoxycholate citrate agar supplemented with antibiotics (‘ASSuT’, see above). The incubation was performed at 37 ± 1 °C for 24 h under aerobic conditions. Samples which failed to grow when plated out directly were examined according to DIN ISO 6579:2002/A1:21,007 Annex D. Blood samples for serology were taken before the 1st (B0) and 2nd (B1) vaccination, before (B2) as well as 6/7 days p.chall. (B3) in the course of the necropsy. After coagulation of the fibrin clot at 20 °C for 4 h, all samples were centrifuged for 10 min at 3500 x g. The sera were then collected and stored at −20 °C until analysis.

All serum samples were tested using a commercially available ELISA kit (Swine Salmonella Ab Test, IDEXX, Hoofddorp, The Netherlands) that detects IgG antibodies against *Salmonella*-specific LPS. This analysis was performed at IVD Diagnostik (Hannover, Germany) according to the manufacturer’s instruction (cut-off < 10 OD%).

Furthermore, all samples were analysed with an optimized in-house WCE ELISA for determining the *Salmonella*-specific IgM, IgA, and IgG immunoreactivity according to the following instruction. The Salmoporc®/Salmoporc STM® vaccine strain was grown overnight for seeding a fresh logarithmic culture which was harvested at an optical density of 0.3 (wavelength = 600 nm; Saphire 2, Microplate reader, Tecan Group Ltd., Männedorf, Switzerland). The bacteria were lysed in 8 M urea at 60 °C for 5 min and centrifuged at 12,000 x g for 10 min. Protein content of supernatant was determined by Bradford assay (SIGMA Aldrich, Deisenhofen, Germany) and 10 μg/ml protein was immobilized onto a 96-well flat-bottomed microplate (Medisorp™, Nunc, Wiesbaden, Germany) at 4 °C overnight. Plates were washed three times with phosphate-buffered saline, 154 mM NaCl, 0.05% Tween (3× PBS-T) and blocked with Superblock™ blocking buffer (Thermo Fisher Scientific, Schwerte, Germany). Swine sera were diluted 12.5-fold for IgM and IgA or 200-fold for IgG determination. After preincubation with soluble *E. coli* proteins, which were prepared from logarithmic culture by lysis with 8 M urea at 60 °C for 5 min and spinning down at 12,000 x g for 10 min, for 1 h at room temperature in order to quench cross-reactivity. Preadsorbed sera were incubated with plate-immobilized STM antigen for 1 h at room temperature in duplicate. Afterwards, antigen-bound IgM, IgG, and IgA antibodies were detected by isotype-specific secondary antibodies against swine IgM, swine IgG or swine IgA (goat anti-swine, all from Bethyl Labs., Montgomery, TX, USA) conjugated to horse radish peroxidase (HRP) and visualized after another washing step by incubating the plates with TMB-E (3,3′,5,5′-tetramethybenzidine) substrate (Moss, Pasadena, MD, USA). Enzymatic reaction was stopped by adding 0.5 M sulphuric acid.

Finally, the optical density (OD) was measured at a wavelength of 450 nm. Colour intensity, which was proportional to the amount of bound antibodies, was calculated into OD% by setting the intensity of a hyper-immune serum (‘hyper-immune serum 97.4’) to 100%. This hyper-immune serum was derived from a pig which was three-fold vaccinated with Salmoporc®/Salmoporc STM® [7] and served as positive control on each plate. The ELISA result is given as OD% relative to the hyper-immune serum.

Statistical analyses were done using a two-tailed Mann-Whitney U test with a level of significance of *p* < 0.05 (SPSS Version 15.0, IBM). The null hypothesis was defined as equal results in the vaccinated and in the control group with respect to the outcome variables. The alternative hypothesis was that the treatment groups will differ. Efficacy of the vaccine (bacteriology) was then assumed if the differences were significant in favour of the vaccination group.

## Results

### Rectal body temperatures

**Fig. 1:**
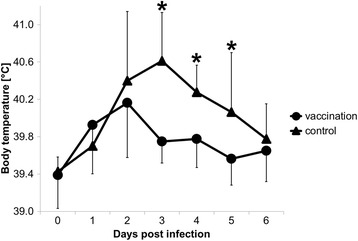
Rectal temperatures during the challenge infection (mean ± standard deviation of each group per day post challenge). Asterisks point to significant differences between the groups (*p* < 0.05)

The rectal temperatures increased after challenge in both groups. However, vaccinated animals showed a lower increase of the rectal temperature and an earlier decline to physiological values compared to controls (Fig. 1). The differences between the groups were statistically significant at days 3, 4 and 5 p.chall. At the end of the observation period (day 6 p.chall.) both groups had nearly equivalent rectal temperatures.

### Clinical examination

Clinical signs during the challenge period were predominantly recorded on days 3, 4, 5 and 6 post infection (p.i.). The vaccinated animals showed significantly lower ‘diarrhoea score’ and ‘clinical score’ on those days, when compared with the control group. Detailed information is given in Table 1.

**Table 1:** Sums of diarrhoea and clinical score per group for each day post challenge

	Day 1	Day 2	Day 3	Day 4	Day 5	Day 6
Diarrhoea score						
Vaccination group	0	0	1	1	0	0
Control group	0	3	4	8	8	8
*p* value	1	0.233	0.233	0.031	0.013	0.013
Clinical score						
Vaccination group	3	4	4	4	0	0
Control group	0	9	11	20	19	19
*p* value	0.1	0.255	0.255	0.003	< 0.001	< 0.001

At the end of the trial (day 6 p.chall.), 5 out of 8 control animals suffered from diarrhoea and all of the controls had at least one elevated value in the ‘clinical score’. None of the vaccinated pigs showed persistent clinical signs at this day.

### Gross pathological examination

Differences between the groups were also detected during the necropsy. Gross pathological findings were confined to the gastrointestinal tract. Catarrhal enteritis of the small intestine was seen in 37.5% of the vaccinated and 87.5% of the control pigs. The latter were additionally suffering from a focal to multifocal diphtheric enteritis in the caecum or colon. None of the vaccinated animals showed diphtheric lesions in the large intestine. Further lesions were not observed.

### Bacteriology

All animals of both groups were found to be infected with the STM challenge strain after challenge. The challenge strain contents in the ileal and caecal mucosa and the ileocaecal lymph nodes were significantly reduced in vaccinated pigs (*p* < 0.05) (Table 2) compared to the non-vaccinated control group.

**Table 2:** STM challenge strain contents in the ileal and caecal mucosa and ileocaecal lymph nodes (in CFU/g tissue: mean ± standard deviation)

		Challenge strain content in lg CFU/g		
Group	Number	Ileum	Caecum	Ileocaecal lymph nodes
Vaccinated	8	4.25 ± 1.25^a^	2.51 ± 2.06^a^	3.57 ± 0.20^a^
Control	8	5.72 ± 0.82	5.85 ± 1.27	4.43 ± 0.37

^a^ *p* < 0.05 (Mann-Whitney U test)

### Serology I (Swine Salmonella Ab test)

**Fig. 2:**
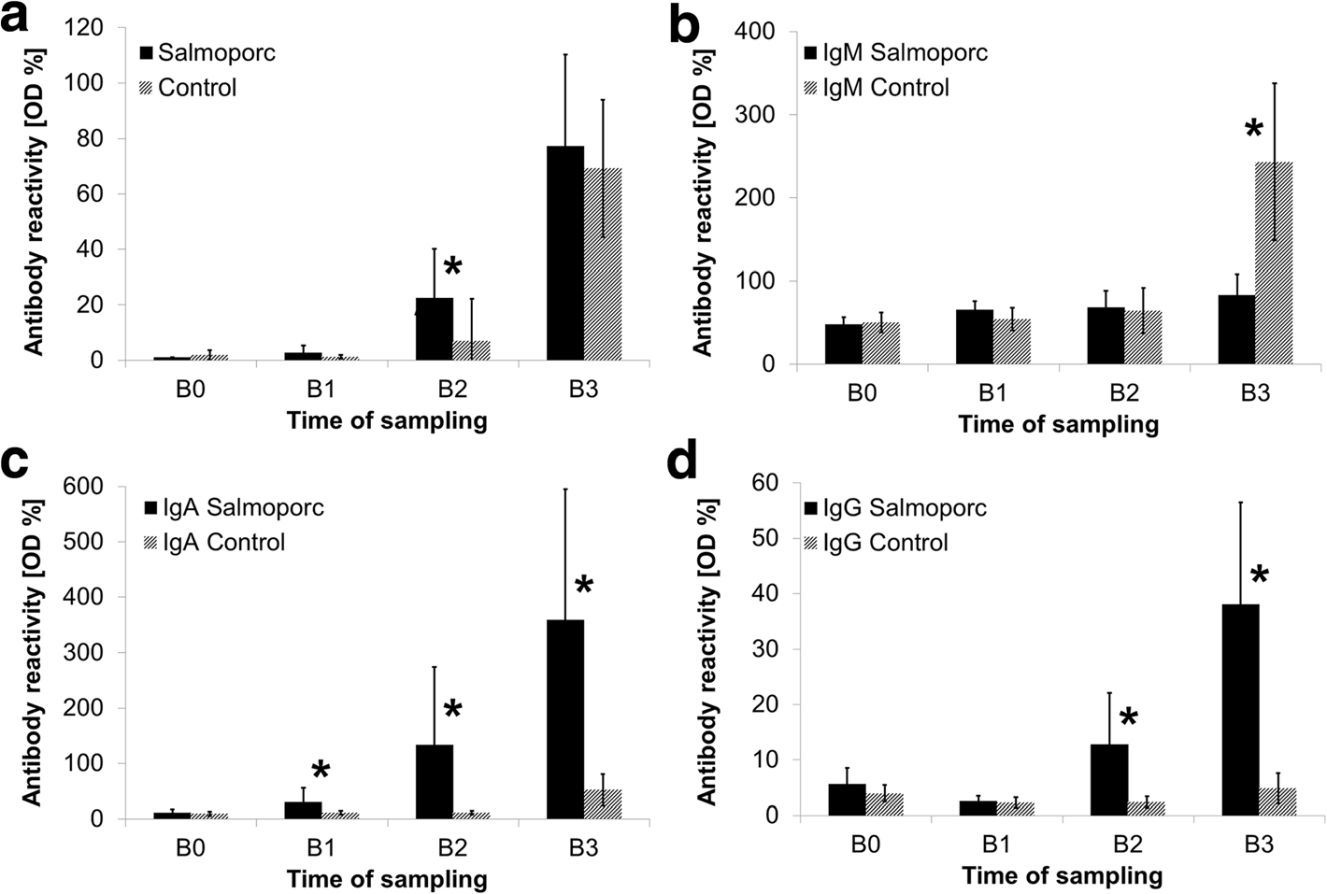
Kinetics of the antibody response after oral vaccination and challenge (OD%, mean ± standard deviation). Asterisks point to significant differences between the groups (*p* ≤ 0.05). B0 = study day 0, B1 = study day 21, B2 = study day 42, B3 = study day 48/49. **a** Following immunization (B2) significant differences between vaccinated and control animals became apparent. At the end of the challenge period both groups exhibit similar values (Swine Salmonella Ab Test). **b** The amount of IgM antibodies was nearly equivalent in both groups until challenge (B0 to B2). At B3 (day 6 post challenge), the IgM OD% values in the control group were found to be significantly increased to both, the IgM level of the control group at B2 and the IgM level at B3 of the vaccinated animals, which showed only a slight increase of STM-specific IgM due to the challenge infection (Ig-isotype specific in-house ELISA). **c** STM-specific IgA showed markedly increasing values in the vaccinated pigs during the vaccination period with a further increase during the challenge. In contrast, the control animals revealed only slightly increasing values during the whole animal trial (Ig-isotype specific in-house ELISA). **d** At the sampling point B0 and B1 statistical differences in terms of STM-specific IgG were neither detectable in the vaccinated nor in the control animals. Prior to and post challenge the vaccinated pigs showed significant higher values compared to the control group (Ig-isotype specific in-house ELISA)

Prior to the first vaccination (B0) all weaners were shown to be serologically negative for *Salmonella* sp. (< 2.25 OD%). At B1 (3 weeks after the 1st vaccination) no antibody response was detectable (Fig. 2). However, 3 weeks after the 2nd vaccination (B2, immediately before the challenge) the vaccinated animals had significant higher antibody levels than the control group. At slaughter (B3) a further remarkable increase of the antibody amounts was detected, but reached similar levels in vaccinated and non-vaccinated animals.

### Serology II (STM-specific IgM, IgA, IgG)

*Salmonella*-specific IgM, IgA and IgG antibody isotypes showed different profiles during the vaccination and the subsequent challenge period. The results are shown in Fig. 2b–d and are specified as proportional constant of the OD%-values at two consecutive times. STM-specific IgM amounts were slightly increasing with the ageing of the piglets during the trial but without any appreciable differences between vaccinated and control pigs. The vaccination had no impact towards the IgM OD% values whereas in response to infection a 3.8-fold increase was detected, as a result of the acute infection. STM-specific IgA values were continuously increasing within the vaccination group starting after the first vaccination (4.4-fold increase from B1 to B2, 1.7-fold increase from B2 to B3). The differences between the groups were shown to be statistically significant from B1 until the end of the trial. In the control group a 5-fold increase of the OD% values was found at days 6/7 p.chall. STM-specific IgG amounts were nearly equivalent in vaccinated and control pigs until B1. Prior to the challenge (B2) the average amount of IgG significantly raised in vaccinated pigs, while the controls showed constantly low OD% levels. After the challenge a further 3-fold increase of the OD% values was detected in the vaccination group when compared with the values at B1. No increase of STM-specific IgG antibodies was observed in non-vaccinated animals.

## Discussion

In this report we describe the immunogenicity of the vaccine Salmoporc®/Salmoporc STM® after challenge with a virulent mSTM DT193 strain including the results of the clinical, gross-pathological and bacteriological examination. Special emphasis was placed on the kinetics of the *Salmonella*-specific antibodies and a differentiated view on the isotypes IgM, IgA and IgG during the vaccination period and the subsequent infection.

Natural infections with STM are often asymptomatic [17–19] but they can also go along with enterocolitis, fever and inanition [20]. In this trial we used a high number of bacteria for inoculation in order to reliably trigger clinical signs. Following challenge infection, clinical signs were observed in both groups, including increased body temperatures, diarrhoea, loss of appetite or reduced general condition. It was clearly shown that the body temperature increased in both groups. However, the vaccinated animals returned significantly earlier to physiological body temperatures than animals of the control group did. The same could also be observed regarding the other clinical signs. Comparable results have previously been reported after challenge with a biphasic STM strain by Selke and colleagues [7].

Differences between both groups were also noted during post-mortem examination, as demonstrated by a significantly reduced prevalence of catarrhal enteritis and the absence of additional diphtheric enteritis in the large intestine of vaccinated animals. Especially the latter lesion is a typical morphological alteration associated with STM infections [20]. Comparable morphological findings were not described by Selke and colleagues [7]. They could only find subtle differences between the groups in terms of a reactive hyperaemia of the intestine. The absence of definite morphological changes could potentially be due to the lower infectious dose in their study (1 × 10^9^ CFU/animal) or differences in the susceptibility of the weaners used in both studies.

During quantitative bacteriological examination we could demonstrate that the bacterial load of the challenge strain was significantly reduced in vaccinated animals. To the best of our knowledge, this is the first report of this particular vaccine that demonstrates efficacy against mSTM in a laboratory trial, as the protective potential against these strains was recently suggested by Davies and colleagues during a large-scale field study [10]. Despite this fact, the protective potential of the vaccine against STM has already been demonstrated in the past [6, 7].

When comparing our results to those reported in the literature with STM [6, 7] it can be concluded that the protective potential of the vaccine is nearly equivalent for both STM and mSTM. However, when considering the efficacy data of previous studies in more detail, it is striking that the bacterial loads in all of the three quantitatively examined tissues in the present report are higher [6, 7]. This might be attributed to the challenge strain. While in the two previous studies biphasic STM DT 104 have been used [6, 7], a virulent DT 193 was used in the present study, which was isolated during a serious human outbreak. This strain might have better colonization capability than the biphasic variants. However, despite the different values of the bacterial load in the mentioned studies and the present report, the reduction of the bacterial load (representing the vaccine’s efficacy) is comparable.

Previoulsly, Methner demonstrated a direct correlation of the bacterial load in the caecum and the shedding of *Salmonella* through faeces in chickens [21]. This relationship has not been proven in pigs so far but is quite conceivable. Therefore, a reduction of the challenge strain content in the ileal and caecal mucosa, which are to be considered as the reservoir for *Salmonella* in pigs, should consequently lead to a reduction of STM shedding.

Vaccination against *Salmonella* induces both humoral and cell-mediated immune mechanism, as shown by Lehmann et al. [11] after vaccination of BALB/c mice with an attenuated *Salmonella* Enteritidis vaccine strain. And both immune mechanisms are crucial during *Salmonella* infections in chickens [22]. In our study, we confined on the humoral immune mechanisms but the cell-mediated immune response during vaccination and infection should likewise be studied in pigs in the future. The strong increase of the base line STM-specific IgG response following vaccination might be the result of T-cell-mediated class-switch recombination from IgM/IgG3 to downstream IgG subclasses (IgG2–6) in *Salmonella*-antigen activated B cells, which would imply the induction of cell-mediated immune mechanisms in the present vaccination model.

We could demonstrate an increase of the OD% values after vaccination using the LPS ELISA (Swine Salmonella Ab test). Nevertheless, the mean OD% values were still below current threshold values, for example the German threshold of 40 OD% [23]. This is in accordance with previous studies using the same vaccine [24, 25].

Within 1 week after infection a further increase of *Salmonella*-LPS specific antibody values was detected in both groups leading to almost the same OD% levels which has also been seen in previous studies [6]. The mean values of the animals were markedly above the threshold and would have been equally judged to be positive for *Salmonella*, independently from the trial group.

By the use of an Ig isotype-specific in-house ELISA we were able to study the kinetics of the relative amounts of IgM, IgA and IgG in pigs after oral vaccination and subsequent challenge. Antibody profiles have been shown by Trepnau and colleagues but without challenge [16]. Furthermore, the authors used a different vaccination scheme as well as an administration of the vaccine via the feeder (possibly reduced dose per animal). This and the fact that the ELISA applied for the differentiation of the immunoglobulin (Ig) isotypes in the previous study (Salmotype® Pig STM-WCE ELISA, Labor Diagnostik Leipzig) is no longer commercially available makes it difficult to compare the published results with those of the present study. Nevertheless, the overall kinetics of IgM, IgA and IgG in the previous report and in our study appear to be very similar.

STM-specific IgA and IgG antibody levels were elevated in response to vaccination with Salmoporc® and showed a further increase during the challenge whereas the OD% levels of the controls did not rise significantly 1 week after challenge. In contrast, STM-specific IgM levels were not elevated by vaccination whereas the challenge, which simulates a field infection, caused a significant increase in non-vaccinated animals within 1 week.

Mucosal pathogens are attacked in the first instance by a local immune response in terms of the secretion of IgA antibodies. This Ig isotype is mainly produced by gut associated lymphoid tissues and transported via luminal epithelial cells into the mucus [26]. Therefore, an activation of this first-line defence by vaccination is a crucial factor for protection against intestinal pathogens. IgA inhibits bacterial motility, circumvents adhesion to epithelial cells and neutralizes bacterial toxins. The first contact of the pathogen with the host proceeds via mucosa and thus bacteria which were neutralized by IgA, were finally hindered from entering host and colonizing organs [27].

Invasive pathogens are adequately combatted by IgG antibodies, which were found to be induced to a lesser degree in our study. Instead, a strong rise of IgA antibodies was detected after vaccination and infection. It has already been shown that innate IgA contributes in a significant manner to defence and recently, a long-lasting IgA memory response was reported to be developed especially after oral immunization [28, 29].

In the present study, a 12-fold increase of *Salmonella*-specific serum IgA in vaccinated animals (compared to controls) was detected. Given that about 30% of serum IgA originates from intestinal production [30] the content of IgA in mucus must be several fold higher than serum levels. A positive correlation between mucosal and systemic IgA levels has been shown [31].

The increase in serum levels of *Salmonella*-specific IgA might assist in eliminating *Salmonella* that evaded the first line mucosal defence and take part in defence of the gut across the hepato-biliary route as secretory IgA [26]. ELISA data show, that despite of the facultative intracellular pathogenic nature of *Salmonella* and the subsequent requirement of cell-mediated immunity to survive infection, humoral immunity mainly due to IgA antibody production decisively contributes to control of infection as previously shown by other authors [32]. Besides the gain of IgA an increase of IgG was present in the vaccinated animals after challenge, as well, which further confirms the immunogenic capacity of the vaccine.

## Conclusion

An oral vaccination with a registered STM live vaccine makes clinical signs induced during a laboratory challenge infection of weaners with virulent mSTM milder. Furthermore, the vaccine is effective in reducing the bacterial count of the challenge strain in the gut and lymph nodes which consecutively would lead to a reduced persistence and shedding of STM/mSTM.

The kinetics of IgM, IgA and IgG were different in vaccinated and control animals. This reveals further information regarding the immune mechanisms being associated with STM infections and vaccination.

## Abbreviations

ASSuT: Ampicillin, streptomycin, sulfamerazine, oxytetracycline
B0-B3: Blood sampling 0 – blood sampling 3
CFU: Colony forming unit
DT: Defined type
ELISA: Enzyme linked immunosorbent assay
HRP: Horse radish peroxidase
IgA: Immunoglobuline A
IgG: Immunoglobuline G
IgM: Immunoglobuline M
LPS: Lipopolysaccharide
mSTM: Monophasic *Salmonella* Typhimurium
OD: Optical density
p.chall.: Post challenge
SE: *Salmonella* Enteritidis
STM: *Salmonella* Typhimurium
TMB: 3,3′,5,5′-tetramethybenzidine
WCE: Whole cell extract
